# How to measure renal artery stenosis - a retrospective comparison of morphological measurement approaches in relation to hemodynamic significance

**DOI:** 10.1186/s12880-015-0086-8

**Published:** 2015-10-12

**Authors:** Malin Andersson, Karl Jägervall, Per Eriksson, Anders Persson, Göran Granerus, Chunliang Wang, Örjan Smedby

**Affiliations:** Center for Medical Image Science and Visualization (CMIV), Linköping University, Linköping, Sweden; Department of Radiology and Department of Medical and Health Sciences, Linköping University, Linköping, Sweden; Department of Nephrology and Department of Medical and Health Sciences, Linköping University, Linköping, Sweden; Department of Clinical Physiology and Department of Medical and Health Sciences, Linköping University, Linköping, Sweden; School of Technology and Health (STH), KTH Royal Institute of Technology, Stockholm, Sweden

**Keywords:** Renal artery stenosis, Computed tomography angiography, Magnetic resonance angiography, Renography, Fuzzy connectedness segmentation, Vessel diameter, Cross-sectional area

## Abstract

**Background:**

Although it is well known that renal artery stenosis may cause renovascular hypertension, it is unclear how the degree of stenosis should best be measured in morphological images. The aim of this study was to determine which morphological measures from Computed Tomography Angiography (CTA) and Magnetic Resonance Angiography (MRA) are best in predicting whether a renal artery stenosis is hemodynamically significant or not.

**Methods:**

Forty-seven patients with hypertension and a clinical suspicion of renovascular hypertension were examined with CTA, MRA, captopril-enhanced renography (CER) and captopril test (Ctest). CTA and MRA images of the renal arteries were analyzed by two readers using interactive vessel segmentation software. The measures included minimum diameter, minimum area, diameter reduction and area reduction. In addition, two radiologists visually judged the diameter reduction without automated segmentation. The results were then compared using limits of agreement and intra-class correlation, and correlated with the results from CER combined with Ctest (which were used as standard of reference) using receiver operating characteristics (ROC) analysis.

**Results:**

A total of 68 kidneys had all three investigations (CTA, MRA and CER + Ctest), where 11 kidneys (16.2 %) got a positive result on the CER + Ctest. The greatest area under ROC curve (AUROC) was found for the area reduction on MRA, with a value of 0.91 (95 % confidence interval 0.82–0.99), excluding accessory renal arteries. As comparison, the AUROC for the radiologists’ visual assessments on CTA and MRA were 0.90 (0.82–0.98) and 0.91 (0.83–0.99) respectively. None of the differences were statistically significant.

**Conclusions:**

No significant differences were found between the morphological measures in their ability to predict hemodynamically significant stenosis, but a tendency of MRA having higher AUROC than CTA. There was no significant difference between measurements made by the radiologists and measurements made with fuzzy connectedness segmentation. Further studies are required to definitely identify the optimal measurement approach.

## Background

Renal artery stenosis (RAS), most commonly due to atherosclerosis, is an important cause of hypertension and accounts for 1–5 % of all hypertension cases in the general population, but when at-risk patients are identified through clinical selection criteria, the estimated prevalence of renovascular-induced hypertension increases to 20–40 % [1]. Currently, there is much debate over how to best treat these patients and the value of percutaneous transluminal renal angioplasty (PTRA) in renal artery stenosis is not completely known [2, 3]. The recently published CORAL study even found that renal artery stenting does not significantly improve clinical outcome [4]. It is therefore important to have reliable diagnostic methods in order to identify patients that could benefit from treatment.

The most commonly available methods to diagnose RAS are computed tomography angiography (CTA), magnetic resonance angiography (MRA), captopril-enhanced renography (CER), captopril test (Ctest), ultrasonography and invasive intra-arterial renal angiography (IARA) [1]. IARA has long been considered the gold standard for diagnosing RAS [5], but it is not an ideal method. It relies strictly on lumen morphology and thus gives only indirect information about the hemodynamic consequences of the stenosis [6]. Besides, the invasive nature also means a risk of severe complications [7–10]. It can sometimes be combined with measurement of the pressure drop through the stenosis, which may indicate more reliably hemodynamically significant RAS [11], although the presence of a catheter in the artery might disturb the measurements [12]. Rapid progress in spatial resolution and image quality has allowed MRA and CTA to replace the invasive examinations as the standard diagnostic methods [1, 13]. Although a major prospective study, conducted between 1998 and 2001 and published in 2004 (the RADISH-trial), concluded that neither CTA nor MRA are reproducible or sensitive enough to replace IARA as the diagnostic gold standard [14], randomized trials comparing CTA and MRA to IARA have shown a sensitivity of 94–95 % and a specificity of 92–93 % for CTA [15, 16], and a sensitivity of 90–100 % and a specificity of 94–98 % for MRA [15, 17].

Renography is a method for functional evaluation of the kidney. The use of angiotensin converting enzyme (ACE) inhibitors, such as captopril, in conjunction with radionuclide renography has been shown to enhance sensitivity and specificity for detecting renovascular hypertension [18, 19]. CER has been reported to have a sensitivity of 87–95 % and specificity of 93–100 % when the reference is whether there is a reduction in blood pressure following intervention [20, 21]. That makes CER a valuable method for detecting hemodynamically significant stenoses. However, in cases with potential bilateral stenosis, as well as in severe renal failure, its value is doubtful [19]. In cases of intermediate test results on CER, the captopril test (Ctest), which includes analysis of the plasma concentration of renin before and after ingestion of captopril, has been applied to strengthen the diagnosis [6].

The advances of computer post-processing techniques enable researchers to obtain detailed quantitative information about different morphological measures of the vessels, such as minimum diameter, diameter reduction, minimum area and area reduction, from CTA and MRA. A diameter reduction of >50 % has been considered hemodynamically significant by many authors [13, 22, 23], whereas others have advocated higher threshold values [24], but this is not necessarily equivalent to a decrease in function of one or both kidneys [6]. There is therefore a need to investigate how to measure RAS on MRA and CTA to find the best predictive measure of the hemodynamic significance of the stenosis. Although Schoenberg et al. have shown that measurements of the area of the stenosis on MRA might be a better predictor of hemodynamically significant stenosis than the diameter [23], there is not much information about how different morphological measures correlate with renal functional tests. Thus, the aim of our study was to compare different morphological measurements describing renal artery stenoses in CTA and MRA images with respect to their ability to predict a hemodynamically significant stenosis as defined by captopril-enhanced renography.

## Methods

### Patients

For this study, the patient material from an earlier study [6] was used. The ethical approval of the acquisition of data for the original publication was given by the regional research ethics committee in Linköping, Sweden (decision nr. 03–412 and M90-05, dated Nov. 4, 2003 and Aug. 10, 2005). Written informed consent was obtained from each patient before examination, and the study was performed in compliance with the Helsinki Declaration. Forty-seven patients of both genders, 18–80 years old, with a screening serum creatinine of 150–300 μmol/L, hypertension and clinical suspicion of renovascular hypertension were consecutively recruited for the study and examined with MRA, CTA, CER and Ctest. Inclusion and exclusion criteria as well as demographic characteristics are described in [6].

### Morphological investigations

The imaging technique for the CTA and MRA examinations has been published previously [6]. The acquisition protocol for CTA specified a voxel size of 0.75×0.5×0.5 mm^3^, and that for MRA a voxel size of 2.0×0.7×0.7 mm^3^. However, in eight of the patients, CTA data with a higher slice thickness of up to 3 mm had to be used, due to incomplete archiving. In one case, CTA data were missing.

**Fig. 1:**
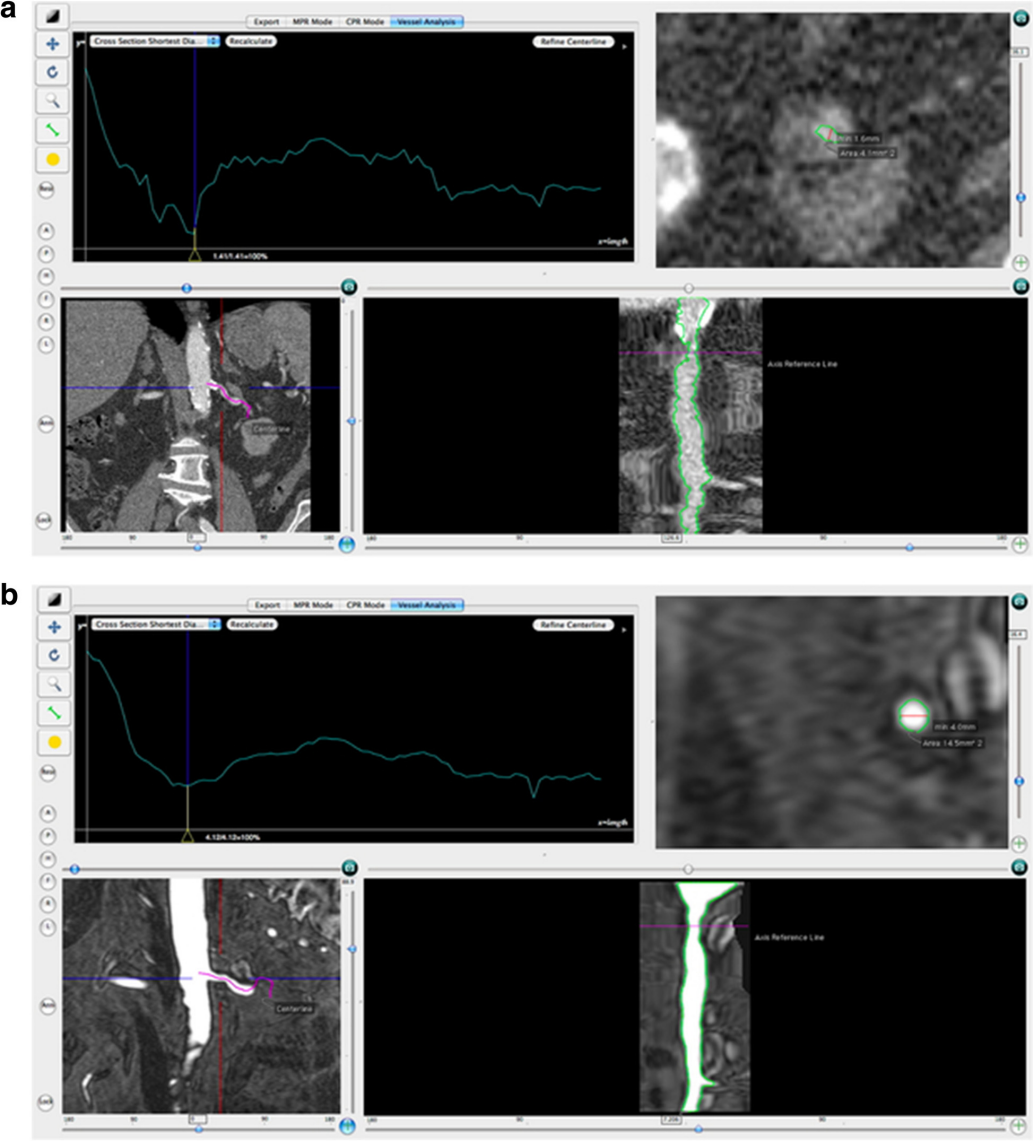
**a**, **b**. A view of the left renal artery in the OsiriX CTA plug-in from CT angiography (**a**) and MR angiography (**b**). In both images, the upper left part is a graph where the vertical axis represents the shortest cross-section diameter in the selected vessel and the horizontal axis represents the distance from the start of the vessel. The upper right part is a cross-section view of the marked position in the graph (in these cases the smallest minimum diameter), the lower left part is a coronal section showing the aorta, the renal arteries and surrounding tissues and the lower right part is the curved plane reformatted view of the chosen left renal vessel. Both modalities show the same stenosis, but it is more accentuated on the CT angiography

Evaluation of CTA and MRA data was made with OsiriX (v.3.6.1 32-bits) [25] and the semi-automated CTA plug-in (CMIV CTA tools) [26] (Fig. 1a and b). The plug-in uses a competing fuzzy-connectedness tree algorithm to segment vessels and extract vessel centerlines [27]. The studied arteries were visualized using curved plane reformatting and the measurements were performed with in a cross-section view of the vessel. The vessel cross-sections were automatically segmented using a thresholding-based level-set method [28]. Some parameters that had to be set in the algorithm were selected after testing and visually assessing the results of alternative settings. The curvature scale was set to 10 to eliminate the effects of noise and keep the vessel contour smooth. A lower threshold (LT) value had to be defined for both MRA and CTA, and for CTA, an upper threshold (UT) was also needed, since calcified plaques have an attenuation exceeding that of contrast-mixed blood.

The LT was set using a grey-scale value in between the value contrast agent and background. The value of contrast agent was determined by measuring the maximum values of ROIs in aorta. The final value is calculated as the mean values from three cross-sections of the aorta (the cross-section of the abdominal aorta giving the greatest mean value and the corresponding slices 10 mm proximal and 10 mm distal to the maximum value). A background grey-scale value was determined by measuring the mean value of ROIs in non-contrast-filled areas. In CTA, four regions of interests (ROIs) were placed in the psoas muscles, and in MRA, three ROIs were placed in the vertebral bodies. For both modalities, the LT was then calculated for each patient with the formula LT = (aorta + 2*background)/3, which visually was the most reliable in a pilot-group of seven patients. The UT for CTA was calculated as the mean value of the maximum value of the cross-section slices of the abdominal aorta and the corresponding maximum values 10 mm proximal and 10 mm distal to the maximum value.

The CMIV CTA tools of the OsiriX software was in most cases able to automatically define the centerline of the vessel. When not possible, or when the automatically created centerline was obviously false, the centerlines had to be drawn manually.

Two segments in each renal artery were measured. The first segment was limited by the aorta and the most proximal bifurcation, while the second segment was defined as the largest branch from the first bifurcation to the second bifurcation. In each segment, the minimum diameter (*MinD*), the minimum area (*MinA*), the maximum diameter (*MaxD*) and the maximum area (*MaxA*) were measured and the relative diameter reduction (*Dred*) was calculated as

$$ Dred=\left(1- MinD/ MaxD\right)\times 100\% $$

and the relative area reduction (*Ared*) was calculated as

$$ Ared=\left(1- MinA/ MaxA\right)\times 100\% $$

To make sure that the *MaxD* and the *MaxA* were not affected by the bifurcation geometry, rules were set prohibiting the readers from measuring the *MaxD* and *MaxA* (i) less than 10 mm from the aorta and (ii) less than 5 mm proximal or distal to a bifurcation. When the rules made it impossible to measure the maximum values (e.g. in cases with very short distances between the branches) they were visually obtained.

The measurements of the renal arteries were made by two medical students, each blinded for the other’s result and for the results of the other modalities. The morphological measures were calculated as the mean value of the two reader’s observations, except for the analysis of the inter-observer agreement. In those cases where only one reader considered a segment measurable, that reader’s measurements were used.

Since two segments were measured in each artery, and each kidney might have more than one artery, several measurements were obtained from each kidney. However, for correlation with the CER + Ctest, only one segment had to be chosen. Three different measurement approaches were used to summarize the results from the two segments of each artery to only one result for the whole kidney (i) the most pronounced stenosis (defined as the segment with the largest *Dred)* in the first segment, or first segments if the patient had accessory renal arteries (referred to as *First*), (ii) the most pronounced stenosis in any segment on one side (*Tightest*), and (iii) the most pronounced stenosis in the main artery (the artery with the largest maximum diameter) (*Main*).

Visual assessment was performed by two radiologists with at least 20 years of clinical experience [6]. The degree of stenosis was defined for both CTA and MRA on a six-grade scale: normal, diameter reduction >10 % but ≤ 30 %, diameter reduction >30 % but ≤ 50 %, diameter reduction >50 % but ≤ 70 %, diameter reduction >70 %, and occlusion. Those results were obtained from the previous study [6].

### Functional investigations

The result from the CER + Ctest was considered to be the reference for defining hemodynamically significant stenoses. A 2-day protocol with a baseline renogram Day 1 and a captopril-stimulated renogram Day 2 was followed [6]. The interpretation of the CER was based on the criteria in [19]. Six patients got an intermediate test result on the CER. In those cases, adding the result of a Ctest made the final interpretation [6]. Two of the 6 intermediate results were judged positive after the Ctest, and 4 were considered negative.

### Statistics

For descriptive purposes, the mean and standard deviation (SD) were used. The inter-observer agreement refers to the difference between the two readers’ results for the same morphological measurement. It was calculated from all vessels judged measurable by both readers. The inter-method agreement refers to the difference between the mean value of the two readers’ CTA measurements and the mean value of the two readers’ MRA measurements. It was calculated at kidney level using the three different measurement approaches described above. The inter-observer and inter-method agreement was described as the 95 % limits of agreements (the mean value of the difference ± 2 SD [29]. In addition, the intraclass correlation (ICC) was computed, treating both cases (kidneys or vessels) and observers as random effects [30]. All these calculations were made with SPSS 19.0.0 (SPSS Inc., Chicago, IL, USA).

To evaluate the morphological methods’ ability to predict hemodynamically significant stenosis as defined by the CER + Ctest, receiver operating characteristic curves (ROC curves) were created for each measurement, as well as for the radiologists’ measurement from the previous study, using the statistical software JMP 9.0 (SAS Inc., Cary, NC, USA). The areas under the ROC curves (AUROCs) of the different measurements in relation to the CER + Ctest were compared using Stata 10.1 (StataCorp, College Station, TX, USA).

## Results

In the 47 patients, where 16 patients had more than one artery per kidney, 104 first segments and 99 second segments of the arteries were considered measurable (by at least one observer) for CTA, and for MRA, 92 first segments and 68 second segments were considered measureable. For CTA, 92 % of the measurable first segments (*n* = 96), and 73 % of the second segments (*n* = 72) were considered measurable by both readers, and for MRA, 90 % of the measureable first segments (*n* = 83) and 67 % of the second segments (*n* = 46) were considered measurable by both readers. The mean values and the standard deviations of the measured segments at artery level are given in Table 1. In general, both the absolute measures (*MinD* an *MinA*) and the relative measures (*Dred* and *Ared*) gave roughly similar values regardless of whether they were computed from CTA or MRA images.

**Table 1:** Descriptive statistics of stenosis measurements (Mean ± SD). Most prominent stenosis defined in three different ways: most pronounced stenosis in proximal vessel segment (*First*), segment with most pronounced stenosis (*Tightest*) and most pronounced stenosis in main renal artery (*Main*)

Modality	Measure	All arteries		Most prominent stenosis for each kidney		
		Segment 1	Segment 2	First	Tightest	Main
CTA	MinD (mm)	2.65 ± 1.46	2.82 ± 1.01	2.93 ± 1.55	2.68 ± 1.44	2.79 ± 1.35
	MinA (mm^2^)	10.56 ± 8.41	9.49 ± 6.08	12.61 ± 9.05	10.86 ± 7.84	11.24 ± 7.55
	Dred (%)	46.7 ± 25.5	24.7 ± 14.7	44.8 ± 26.2	48.4 ± 24.9	46.3 ± 23.2
	Ared (%)	58.4 ± 25.5	36.3 ± 17.6	55.5 ± 26.0	59.3 ± 24.0	57.7 ± 22.9
	*n*	96	72	68	68	68
MRA	MinD (mm)	2.88 ± 1.76	2.78 ± 1.37	3.06 ± 1.74	2.62 ± 1.64	2.64 ± 1.62
	MinA (mm^2^)	11.63 ± 9.49	9.66 ± 7.01	12.90 ± 9.61	10.54 ± 8.53	10.58 ± 8.49
	Dred (%)	45.4 ± 30.8	33.1 ± 25.5	43.6 ± 29.3	50.2 ± 28.2	49.2 ± 27.6
	Ared (%)	55.8 ± 29.4	44.1 ± 25.9	53.3 ± 28.1	60.0 ± 26.0	59.3 ± 25.5
	*n*	83	46	68	68	68

Our material consisted of 47 patients, hence 94 kidneys. Only patients who had all three investigations (MRA, CTA and CER + Ctest) were included. Eleven kidneys got a positive result on the CER + Ctest, and the kidneys contralateral to the positive results were excluded since the method is unable to give useful information in such cases. Four kidneys were excluded because their vessels were non-measurable on either CTA or MRA. One of the kidneys that were non-measureable on MRA had a positive result on the CER + Ctest and the contralateral kidney was thereby excluded. Thus, the analyses were made on 68 kidneys, where 11 kidneys (16.2 %) had a positive result on the CER + Ctest and 57 kidneys (83.8 %) had a negative result. The mean values and standard deviations of the vascular morphology measures for the 68 kidneys using the three different measurement approaches are found in Table 1. Again, no obvious difference was found between CTA and MRA. The smallest *MinD* and *MinA* and largest *Dred* and *Ared* were found for both CTA and MRA in the *Tightest* approach, while the largest *MinD* and *MinA* and smallest *Dred* and *Ared* were found with the *First* approach.

### Inter-observer agreement

**Fig. 2:**
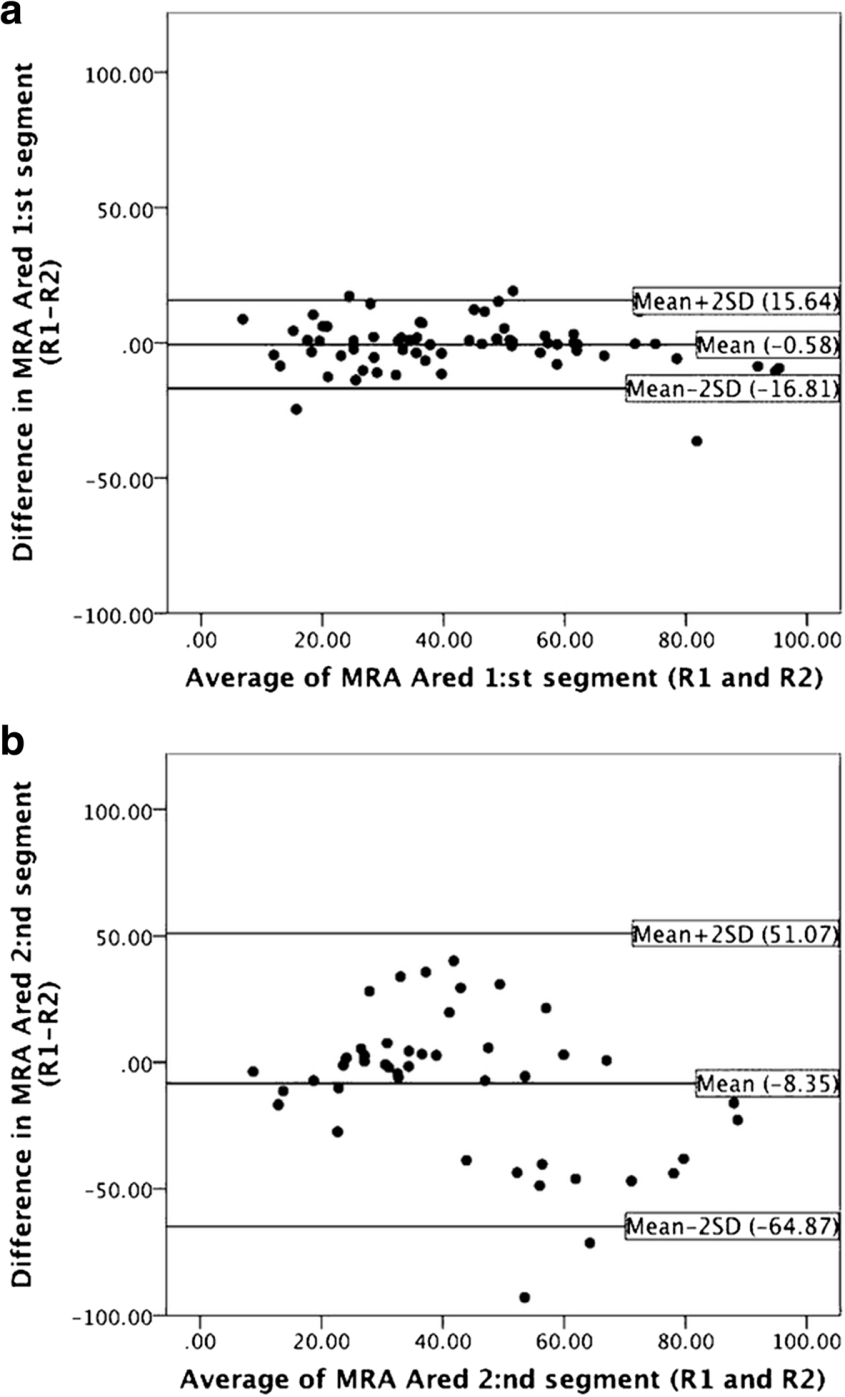
**a**, **b**. Bland-Altman plots showing the agreement between the two readers (R1 and R2) for *Ared* in MR angiography for the first (**a**) and second (**b**) segment. The agreement is highest for the first segment as it has the narrowest limits of agreement

The inter-observer differences in the measurements from CTA and MRA are shown in Table 2. There was excellent agreement for all measurements in the first segment (ICC values between 0.91 and 0.98), while the agreement was lower for the second segment (ICC values between 0.48 and 0.87). For each measurement, the agreement was significantly higher for the first segment than for the second segment. On CTA and on the first segment in MRA there seems to be no systematic under- or overestimation by any reader, but on the MRA of the second segment, the negative mean differences between reader 1 and reader 2 imply that reader 2 measured more severe stenoses than reader 1. Figure 2 shows Bland-Altman plots illustrating the difference in agreement between the two segments, here shown for MRA *Ared.*

**Table 2:** The inter-observer agreement of morphological measures described as the mean difference Reader 1 – Reader 2 and the 95 % limits of agreement (mean –2SD; mean + 2SD), as well as the intraclass correlation and its 95 % confidence limits, *n* = 68

Measure	Segment 1		Segment 2	
	Mean difference (95 % limits of agreement)	Intraclass correlation (95 % confidence limits)	Mean difference (95 % limits of agreement)	Intraclass correlation (95 % confidence limits)
CTA MinD (mm)	−0.11(−1.09; 0.86)	0.97 (0.95–0.98)	−0.11(1.57; 1.35)	0.86 (0.78–0.92)
CTA MinA (mm^2^)	0.08(−5.68; 5.83)	0.97 (0.95–0.98)	−0.48(−9.27; 8.31)	0.87 (0.80–0.92)
CTA Dred (%)	2.46(−22.08; 26.99)	0.94 (0.91–0.96)	2.40(−31.10; 35.90)	0.57 (0.32–0.73)
CTA Ared (%)	−2.03(−33.01; 28.94)	0.91 (0.86–0.94)	−1.91(−41.02; 37.19)	0.64 (0.42–0.78)
MRA MinD (mm)	−0.07(−1.16; 1.03)	0.97 (0.96–0.98)	0.15(−2.32; 2.63)	0.70 (0.47–0.84)
MRA MinA (mm^2^)	−0.23(−8.25; 7.79)	0.95 (0.93–0.97)	0.40(−10.52; 11.32)	0.76 (0.57–0.87)
MRA Dred (%)	0.97(−17.65; 19.59)	0.98 (0.96–0.98)	−6.90(−64.87; 51.07)	0.48 (0.08–0.71)
MRA Ared (%)	−0.58(−16.81; 15.64)	0.98 (0.97–0.99)	−8.35(−64.59; 47.90)	0.54 (0.19–0.74)

### Inter-method agreement

The inter-method analysis was made using the 68 vessels that were compared with the CER + Ctest. The result of the statistical analysis of the agreement between CTA and MRA is shown in Table 3. Although all measurements have an excellent agreement, there is generally a higher agreement for the measurements of the area than for the measurements of the diameter, with the highest ICC being found for *MinA* of *First*. For all measurement approaches, *Dred,* which is the traditionally most used measure, has the lowest agreement. When comparing the three measurement approaches, the agreement is generally highest for *First* and lowest for *Tightest.*

**Table 3:** The inter-method agreement of morphological measures described as the mean difference of Reader 1 – Reader 2 and the 95 % limits of agreement (mean –2SD; mean + 2SD), as well as the intraclass correlation and its 95 % confidence limits. *n* = 68

Measure	Mean difference (95 % limits of agreement)	Intraclass correlation (95 % confidence limits)
MinD first	−0.14(−2.30; 2.03)	0.88 (0.80–0.93)
MinA first	−0.29(−9.85; 9.27)	0.93 (0.89–0.96)
Dred first	1.25(−40.31; 42.81)	0.84 (0.74.0.90)
Ared first	2.12(−29.95; 34.20)	0.90 (0.84–0.94)
MinD tightest	0.06(−2.30; 2.43)	0.83 (0.73–0.90)
MinA tightest	0.32(−10.46; 11.09)	0.88 (0.81–0.93)
Dred tightest	−1.83(−44.20; 40.53)	0.81 (0.70–0.88)
Ared tightest	−0.63(−33.58; 32.32)	0.88 (0.80–0.93)
MinD main	0.15(−2.04; 2.34)	0.84 (0.75–0.90)
MinA main	0.66(−9.55; 10.87)	0.89 (0.82–0.93)
Dred main	−2.93(−42.29; 36.42)	0.83 (0.72–0.89)
Ared main	−1.60(−32.80; 29.60)	0.89 (0.81–0.93)

### Comparison of morphological and functional investigations

There was a tendency of MRA having higher predictive ability (AUROC) than CTA (Table 4); however, there were no significant differences between them. The highest AUROC of all measurements made with the computer-assisted vessel analysis tool was found for *Ared* on *Main* in MRA (0.91) and the highest value for CTA was found for the *Dred* on *Main* (0.88). The lowest AUROC was found for *MinA* and *Ared* on CTA in *Tightest* (0.81), while the lowest AUROC for MRA was *Dred* in *First* (0.84). For the radiologists’ visual assessment on a six-grade ordinal scale, the AUROC were 0.90 for CTA and 0.91 for MRA. The absolute measures had highest AUROC in *First* while the relative measures had the highest AUROC in *Main. Ared* had higher AUROC than *Dred* for MRA, but lower on the CTA. There were no significant differences between any of the AUROC values.

**Table 4:** Relationship between the morphologic measures and the CER + Ctest expressed as the area under the ROC curve (95 % confidence interval), *n =* 68

Measure	First	Tightest	Main
CTA MinD	0.86 (0.77–0.95)	0.83 (0.71–0.94)	0.86 (0.76–0.96)
CTA MinA	0.86 (0.77–0.95)	0.81 (0.70–0.93)	0.84 (0.74–0-95)
CTA Dred	0.85 (0.76–0.95)	0.84 (0.73–0.95)	0.88 (0.78–0.98)
CTA Ared	0.83 (0.71–0.95)	0.81 (0.67–0.95)	0.83 (0.70–0.97)
MRA MinD	0.90 (0.81–0.99)	0.87 (0.77–0.98)	0.88 (0.78–0.99)
MRA MinA	0.90 (0.80–0.99)	0.87 (0.76–0.98)	0.88 (0.77–0.99)
MRA Dred	0.84 (0.70–0.99)	0.88 (0.78–0.98)	0.89 (0.80–0.99)
MRA Ared	0.86 (0.73–0.99)	0.89 (0.81–0.99)	0.91 (0.82–0.99)

## Discussion

A central finding in this study is the excellent ICC values for the inter-method agreement and for the inter-observer agreement of the first segments, which indicate good reproducibility for measuring RAS with the computer-assisted vessel analysis tool. The high reproducibility is probably due to the low influence of individual arbitrariness because of the highly automated work-flow. The formula for the threshold values, the well-defined measuring rules, the automatically generated vessel centerlines and the use of the graph in the CMIV CTA plug-in all make it easy to extract the minimum and maximum values.

As the differences in AUROC were not significant, it cannot be concluded whether measurements from the software tool or visual assessments by radiologists are the best predictors of a hemodynamically significant stenosis. In spite of a tendency for AUROC in general being higher for the visually assessed measurements than for the computer-assisted vessel analysis tool, there were no, or only slight, differences when the best measures with the software on MRA and CTA were used in comparison with the visually assessed measurements (0.91 vs. 0.91 for MRA and 0.88 vs. 0.90 for CTA). It is, however, interesting to notice that users without extensive training (medical students) using the computer-assisted vessel analysis tool were able to attain AUROC values very close to those of the visual assessments of experienced radiologists. Since the influence of individual arbitrariness is minimized when measuring renal arteries with the standardized algorithm in a much automated process, the level of education may be a minor problem for obtaining adequate results. Also, with an excellent inter-observer agreement for the first segments and an excellent inter-method agreement, the reproducibility for the method appears satisfactory. The good reproducibility and AUROCs fairly similar to the radiologists’ measurements suggest that computer-assisted vessel segmentation is a promising method for analyzing RAS, even though definite conclusions about the clinical utility cannot be drawn.

As mentioned earlier, the inter-observer agreement was excellent for the first segments, but it was considerably lower for the second segments. The inter-method agreement was also lower for the second segment as the highest ICC-value was found in *First*, which is the only measurement approach that excludes the second segments. One reason might be that the second segment was defined as the largest branch from the first bifurcation to the second bifurcation, and in some cases it was not obvious which branch was the largest. This might lead to the two readers measuring different segments, which could lead to a lower agreement. One might speculate that if the second segments were excluded, the measurements would be more accurate and therefore have a higher correlation with the functional evaluation. However, the highest AUROC was found in *Main*, which includes the second segment. This suggests that despite being more difficult to measure, the most relevant measurements are those that include the second segment (*Main* and *Tightest* rather than *First*).

Since there were no significant differences between any of the morphological measures in their ability to predict a hemodynamically significant stenosis, no definite conclusions can be drawn. However, a tendency of MRA having higher AUROC than CTA was found, but further studies are required to verify this finding. One explanation might have been that one method systematically measured more distinct stenoses than the other; for instance, gadolinium-enhanced MRA is considered to overestimate the degree of RAS [23]. However, since Table 4 shows that there are no systematic differences between CTA and MRA, this does not seem as a likely explanation of our findings. Our impression is that CTA yielded the best spatial resolution but had inferior image-to-noise characteristics, which may be related to X-ray attenuation in surrounding abdominal organs. This might be a possible explanation for tendency to lower AUROC for CTA than for MRA. The lack of significant differences between the morphological measures might indicate that there are no major differences between them, but the fact that we found that *Ared* has the highest AUROC for MRA is in line with the previous result from Schoenberg et al. who have suggested that the cut-off value for a hemodynamically significant renal artery stenosis can be better defined on MRA by measuring the area of the stenosis instead of the diameter [23]. In the coronaries, Zhang et al. found area reduction to be superior to diameter reduction in predicting numerically computed fractional flow reserve [31]. For CTA, on the other hand, the area seems to have a lower concordance with CER + Ctest than the diameter. There is not much information available on the reliability of the different measures on CTA, and, for both modalities, due to the lack of significant results, no definite conclusions can be drawn.

The purpose of applying three different measurement approaches was to investigate whether it is of any importance which segments are included in the measurements. Of the three approaches, the maximum AUROC for both CTA and MRA was found for *Main*, and the minimum AUROC was found for *Tightest. Tightest* and *Main* are fairly similar with the only difference being that *Tightest,* in cases of more than 1 artery per kidney, also takes the smaller arteries in account. This result might indicate that stenoses in the main arteries are more crucial than stenoses in smaller arteries, or in other words, a stenosis in a small accessory artery may not affect whole kidney function enough to be detected by CER + Ctest. Another possible explanation is that the larger main arteries are easier to measure as their larger size lead to more accurate measurements.

A common reference when calculating *Dred* and *Ared* seems to be the North American Symptomatic Carotid Endarterectomy Trial (NASCET) criteria [32], which uses a normal vessel segment distal to the stenosis instead of the widest part of the segment, thereby excluding a possible post-stenotic dilatation. Although initially developed for the carotid arteries, it has also been used for measuring the renal arteries [33]. A difficulty with that approach might be that a post-stenotic dilatation extends all the way to the next bifurcation. Another used reference is the diameter and area of the main renal artery immediately proximal to the first bifurcation [34]. In this study, the maximum diameter and the maximum area of the segment were used as reference when calculating the relative reductions (*Dred* and *Ared*), in order to get a more standardized and thus more reproducible process. A limitation of our method is that the *MaxD* and *MaxA* might be measured in a post-stenotic dilation, which might lead to an overestimation of the degree of the stenosis. On the other hand, the presence of post-stenotic dilatations speaks in favor of hemodynamically significant stenoses, and if they were considered when calculating *Dred* and *Ared,* the correlations with CER + Ctest might be better. Another limitation is the fact that we measured stenosis using diameters and cross-sectional areas only, thus ignoring the length of the stenosis, which might also influence arterial blood flow.

When measuring vessels in a 3D dataset, the voxels should be as small as possible and preferably close to isotropic, i.e. have the same size in all three dimensions. With the scanners we had available, this was not feasible, and the MRA voxels (2.0×0.7×0.7 mm^3^) were larger than the CTA voxels (0.75×0.5×0.5 mm^3^). The comparatively thicker slices for MRA may have influenced the results for MRA, in particular for *Dred*, which depends heavily on a single diameter measurement. The poorer inter-observer agreement in segment 2 as compared to segment 1 (Table 2) may also be related to the limited spatial resolution.

The ideal method for determining the hemodynamical significance of the stenosis in a clinical context would be to evaluate the outcome of interventions such as PTRA, complemented with information about loss of renal parenchyma. However, the effect of the intervention is confounded by concurrent medical therapy, patient-related preferences and costs [6]. We therefore considered it impractical to use the therapeutic outcome as the reference method. IARA combined with measuring the pressure drop over the stenosis would have been an interesting reference method since it has been shown to correlate with the release of renin behind the stenosed kidney, thus a good indicator of whether the stenosis contributes to hypertension or not [11]. However, it has the obvious drawback of being an invasive method and it is shown to be associated with a risk of severe complications, including cholesterol embolization, pseudoaneurysm, hematoma [7, 9], arteriovenous fistula and contrast-induced acute renal failure [8, 10]. The presence of a catheter in the artery might also alter the arterial fluid mechanisms, and thereby disturb the measurements; however, using a smaller pressure-sensing guidewire than the conventional catheter might reduce the disturbances [12]. The risks of IARA with measurement of the pressure gradient made it ethically unacceptable to perform an invasive catheterization in those patients where the non-invasive diagnostic methods did not indicate a stenosis, and it was therefore not included in the study protocol. For the same ethical reasons, invasive measurement of renin concentrations in the renal veins [35] was also excluded. Duplex ultrasonography would be another potential reference method. It has the advantage of providing both morphologic and functional assessment of the renal arteries, and it is a fast and inexpensive method, but it is very dependent on the skill of the observer. There are also difficulties in obtaining reproducible results from different sites [18, 19]. Ultrasound was therefore not considered an optimal reference method for the study.

CER + Ctest was chosen because it is a well-known method widely accepted for determining the hemodynamic significance of the stenosis [36]. It has been reported to have good sensitivity (87–95 %) and specificity (93–100 %) for detecting RAS when the reference is whether there is a reduction in blood pressure following intervention [20, 21], suggesting that CER + Ctest is a good predictor of the hemodynamic aspect of the stenosis. Its non-invasive nature also makes it a less harmful procedure compared to IARA. As the aim of the study was to investigate how different morphological measures of a stenosis correlate with the hemodynamic significance, we considered CER + Ctest to be the most suitable reference method. However, the question might be raised whether CER + Ctest is the optimum reference method in this study as the material included patients with moderately reduced renal function (serum creatinine of 150–300 μmol/L), where CER is less sensitive [37]. The sensitivity for detecting RAS with CER in patients with slightly to moderately reduced renal function has been reported between 75 and 87 % [38]. Pedersen [36] draws the conclusion that ACE inhibitor renography seems able to diagnose RAS in patients with reduced renal function as long as the renal function is not severely impaired. Therefore, CER + Ctest was still considered the most suitable reference method for our material despite the moderately reduced renal function of the included patients.

The lack of significance of the results in our study could be explained by the small number of cases, especially the few positive cases (11 kidneys) on CER + Ctest. Such a small number of positives may give random variations a big impact and therefore results in wide confidence intervals. Because no outlier analysis was made, a few extreme measurements might have severe impacts on the results.

## Conclusion

No significant differences were found between different morphological diameter and area measures in their ability to predict hemodynamically significant RAS. However, there is a non-significant tendency of MRA being a better predictor than CTA with the area reduction in the main artery as the best measurement. Automated measurements with the interactive vessel segmentation software showed very good reproducibility and only slightly lower validity than the visually assessed measurements in predicting the outcome of CER + Ctest, suggesting that this segmentation tool might be useful in the diagnosis of RAS. Nevertheless, the diagnostic value of morphological measures of renal artery stenosis is still incompletely known, and further studies using larger materials and scanners permitting higher spatial resolution are needed.
